# Kano-QFD-based analysis of the influence of user experience on the design of handicraft intangible cultural heritage apps

**DOI:** 10.1186/s40494-023-00903-w

**Published:** 2023-03-23

**Authors:** Jia Li, KieSu Kim

**Affiliations:** 1 School of Journalism and Communication, Sichuan International Studies University, Chongqing, 400031 China; 2 School of Design, Silla University, Busan, 46958 Korea

**Keywords:** UX design, Handicraft ICH apps, Kano-QFD, User needs, Design requirements

## Abstract

Mobile internet and digital technology provide new media platforms for the dissemination of handicraft intangible cultural heritage (ICH). Despite extensive research into the effect of user experience (UX) on handicraft ICH applications (apps), there is little quantitative research on the relationship between user needs and app design requirements. This study employed semi-structured interviews, online questionnaire surveys, and expert evaluation to understand and rank user needs and design requirement influencing factors. A quantitative analysis using the Kano-QFD approach revealed the different effects of twelve user needs and twenty-three design requirements on handicraft ICH app design. Following the establishment of a relationship matrix between user needs and design requirements, the app design requirements were ranked according to importance. Furthermore, a concept–category–application UX app design framework was constructed based on an analysis of the importance of design requirements. The concept layer formed the theoretical basis for the UX app design, the category layer represented the different facets of the UX design, and the application layer provided specific suggestions on the importance of app design. The eye-movement experiment and System Usability Scale were used to test the UX effects of a sample handicraft ICH app, verifying that the UX design framework was reliable. The framework provides methodological reference for the UX design of other ICH digital products.

**Supplementary Information:**

The online version contains supplementary material available at 10.1186/s40494-023-00903-w.

## Introduction

Handicraft intangible cultural heritage (ICH) is living artisan culture [1] which, according to UNESCO, is “the most tangible manifestation of ICH” [2]. Ensuring the continuation of practices and skills requires supporting skilled inheritors to produce handicrafts and to pass their skills and knowledge to others, especially within their own communities. Increasingly, multiple forms of ICH are being more widely inherited through various media that actively disseminate handicraft knowledge and skills and market handicraft products. In the era of mobile internet, handicraft ICH practitioners use mobile networks, smart devices, and digital technologies to create online and offline platforms to broaden public understanding and recognition of their work [3–5]. Understanding use of the internet and new media platforms to enhance public recognition and experience of traditional craft culture is important in the protection and continued inheritance of ICH [6, 7].

In the era of fifth-generation technology (5G), smartphones and mobile terminals have become the primary means of information exchange, and a vast range of mobile apps have been developed to meet different user needs and usage scenarios. In China, handicraft ICH apps are developed and operated by craft creators, industry organizations, and network developers. Different apps focus on creator content sharing, entertainment, and sales and marketing. Digital technologies, such as games, virtual reality (VR), augmented reality (AR), and blockchain, allow for engagement and interaction with, among others, digital collections and virtual exhibition halls, and the use of social media, mobile short video and other intuitive and interactive new media increase and expand access to handicraft ICH. The diversified form and content, real-time updates, efficient dissemination, and strong interactivity of mobile apps mean that they have become important network platforms for the protection and dissemination of ICH [8, 9]. Mobile apps allow users to quickly and easily perceive and experience traditional craft culture via their mobile devices. Therefore, in order to enhance interactive user experience, it is necessary to systematically and scientifically analyze user functional needs and emotional experiences [10]. In this way, user satisfaction and pleasure within the dynamic digital environment can be enhanced [11], thus encouraging greater use and uptake of these apps, which will further contribute to the dissemination, inheritance, and protection of handicraft ICH.

The aim of this research was to establish how to translate user experience (UX) needs into app design requirements, in order to enhance UX. We conducted semi-structured interviews with handicraft ICH app users, supported by literature and expert opinion, to establish user needs in order of importance. These data were analyzed using the combined user requirement acquisition tool (Kano model) and the quality function development (QFD) method (Kano-QFD), to rank the importance of user requirement weights and their design requirements. The results were then tested by trialing the UX of a newly-designed handicraft ICH app on users and non-users. We found that the Kano-QFD method offers a more nuanced approach to the UX design of ICH digital products.

## Literature review and theoretical basis

### ICH apps UX: three research perspectives

The concept of user experience (UX) was first proposed by scholars such as Donald Norman in the field of industrial design [12]. This concept has expanded within the growing field of UX research [13, 14]. In the experience economy, “user-centered” is a logical starting point for the communication of cultural innovation, emphasizing the self-perception of the user when interacting with products [15]. As such, UX has become a key indicator for measuring product quality [16]. The representation of digital products through apps has become a deeply integrated aspect of daily life. In the area of ICH, the design of apps has important practical significance for expanding user experience of ICH culture and for promoting the more diversified inheritance of traditional skills. At present, ICH app UX research focuses mostly on three areas: emotional experience, functional experience, and environmental experience.

The emotional experience perspective explores user attitudes and user behaviors, and analyzes the sensory needs, social needs, aesthetic perceptions, and aesthetic imaginations of users. Hincapié et al. [17] utilized two sets of comparative experiments to test user learning issues when activating cultural heritage technical apps. These app platforms, based on GPS and augmented reality technologies, aim to improve participants’ knowledge acquisition and interactions with urban cultural and architectural heritage. McGookin et al. [18] conducted a 5-day evaluation of 45 participants to investigate user behavior of a cultural heritage app for Seurasaari Island in Finland, focusing on how low-immersion technology exposed participants to cultural heritage. Xu and Sun’s [19] structural equation model analysis established that outcome expectations, ease of use expectations, the intervention of others, and promotion conditions have a significant impact on public willingness to engage with and utilize handicraft ICH digital information, although moderating variables such as gender, age, and educational background have a certain impact. Wu [20] argued that the ‘pain points’ of ICH apps should be resolved through a quantitative analysis of user needs, changing the role of designers, changing browser reading habits, regular maintenance, and timely updates.

The functional experience perspective focuses on the product usability of ICH apps, including content creation, technical processing, media form, and interface design. Arrighi et al. [21] investigated the digital reconstruction of an Australian heritage building, Victoria Theatre, focusing on the availability of cross-media, virtual reality technology that takes a viable interaction approach to digital heritage. Vrettakis et al. [22] developed Narralive, a web-based, storyboard editor, with a mobile playback app version, that they hoped would change the way audiences engage with cultural heritage. Ren et al. [23] conducted quantitative analysis of experimental data to calculate the utility value of each design element, allowing them to study user preferences for the interactive interface of ICH apps and to establish app interface design principles. Chen et al. [24] applied cognitive graphics theory to analyze an interaction model and interaction design principles of traditional handicraft apps, using Chinese Guangcai porcelain as the prototype to carry out design practice.

The environmental experience perspective focuses on the usability and scene factors of ICH app products and services, including the sensory environment, the media interaction environment, and evaluation indicators. De Paolis et al. [25] developed an immersive virtual reality app by collecting tangible and intangible cultural heritage from a castle in Salento, Italy. They tested it with different user samples, and obtained feedback from users on the immersive experience of the app. Zhang and Peng [26] drew on Garrett’s user experience element model to construct an evaluation index and design model of an ICH app and developed a Chinese twenty-four seasons app by analyzing a logical relationship between the internal and external environments and the system design principles of the design model. Xu and Lu [27] employed service design thinking and the principle of “community and custom service” to design the Seeking Place handicraft ICH app to support the creation of ICH handicraft culture and derivatives online and offline. Huang et al. [28] proposed and verified a four-part evaluation index system of mobile apps that focuses on application platform, user experience, visual foreground, and network background.

These three approaches to the user experience of ICH apps focus on users (emotional experience), products (functional experience), and usage environment (environmental experience). A great deal of extant literature addresses user preferences, digital technology adoption, evaluation metrics, and usability practices. However, less attention has been paid to user aesthetic emotions and the media interaction experience of ICH apps. In particular, the metrics related to UX are mostly general and limited, and UX surveys and quantitative analysis that focuses on handicraft ICH apps are even rarer. In addition, in user attitudes and behaviors research, most of the empirical results of user needs are copied to micro-objects such as app information architecture and interface design, while disregarding the transformation of user needs factors in design applications. This disregard leads to apps that are difficult to understand and use, resulting in poor user experience.

### Kano-QFD

**Fig. 1:**
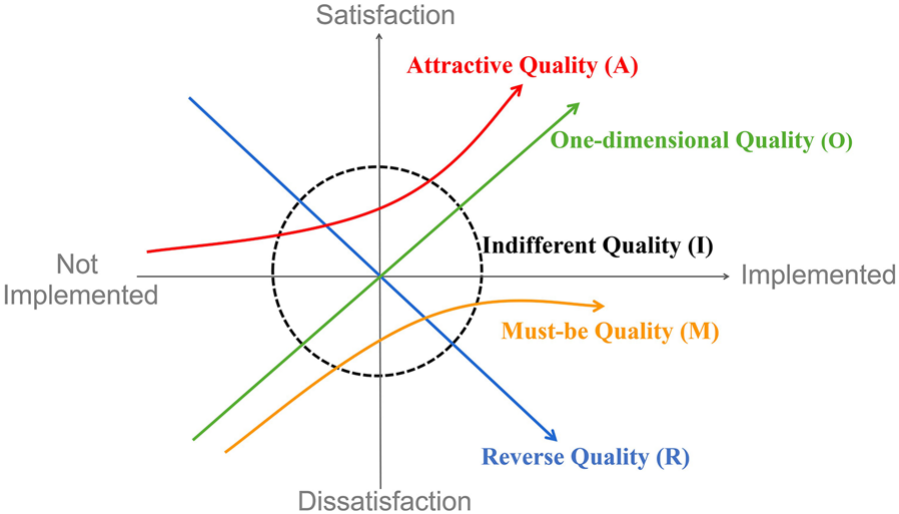
Kano model

The Kano model, developed in 1984 by Noriaki Kano, is a method for measuring customer satisfaction and informing product development. The model classifies and prioritizes how user needs are mined [29–31] according to five categories: (1) attractive qualities (A) that give satisfaction when present but do not cause dissatisfaction when not present; (2) one-dimensional qualities (O) that give satisfaction when present and dissatisfaction when not present; (3) must-be qualities (M) that are expected and taken for granted by users; (4) indifferent qualities (I) that are neutral and cause neither satisfaction nor dissatisfaction; and (5) reverse qualities (R) that result from a high level of development and may cause satisfaction in some users and dissatisfaction in others (see Fig. 1). The indifferent quality (I) does not affect user satisfaction and the reverse quality (R) is dependent on individual user tastes for more or less technology. Therefore, in this study, these two items do not need to be calculated for user satisfaction.

Quality function development (QFD) is another method of Japanese origin that is used to correlate user needs with product design. An integral part of the QFD method is the House of Quality, a method of quantitative analysis that translates user needs (‘Whats’) into distinct design requirements (‘Hows’) [32–34]. The House of Quality uses this quality matrix to decompose user needs into each stage of product design and to promote cooperation among the different departments associated with the product to ensure product design quality. The House of Quality comprises a left wall, a roof, and a room: the left wall consists of user demands and their importance, the roof is the design requirement of the app, and the room is the relationship matrix between the user demand and the design requirement.

This study combines the Kano model with the QFD method (Kano-QFD) to obtain user needs, identify key design factors, and implement the app design. The Kano-QFD method has previously been applied to product design, interaction design, and service experience design and it has theoretical and practical dimensions.

From a theoretical perspective, the Kano model has the advantage of being able to sort out user requirements, while the QFD method aids planning and design. Therefore, researchers combine the advantages of both to understand user needs attributes, design requirements transformation, and design framework construction. Matzler and Hinterhuber [35] have investigated different user needs based on the Kano model and have demonstrated how these different attributes can be translated into product design factors using the QFD method. Tan and Shen [36] incorporated the Kano model into the planning matrix of the QFD method and proposed an approximate transformation function to adjust the improvement rate of each customer attribute, adjusting the customer’s original priority accordingly. Based on the Kano-QFD method, Chaudha et al. [37] proposed a method to adjust the importance level of product design or service attributes based on customer survey data. Afshan and Sindhuja [38] explained the role of the QFD method in product development and design framework construction and then demonstrated how combining it with the Kano model could improve customer satisfaction.

From a practical perspective, Kano-QFD has been repeatedly shown to be effective in everything from industrial to digital products, and from usability design to experiential design. Hashim and Dawal [39] surveyed 336 students in a Malaysian secondary school and then employed Kano-QFD to analyze the design of student classroom furniture in order to meet the ergonomic needs of the students. Yadav et al. [40] combined the QFD method with a fuzzy Kano model to classify the aesthetic attributes of cars in order to guide improvements to their shape design. Zhu et al. [41] demonstrated that Kano-QFD was an effective method for user experience design by designing a music app interface for smart phones for older people. Wei et al. [42] constructed a Kano-QFD usability design framework and realized the usability design of the Cloud Pet app according to its information architecture, interaction prototype, and interface design. Tontini [43] designed a draft beer mug as an object and integrated the Kano model into the QFD analysis, focusing on the needs and real experiences of consumers during the development of the mug.

Most Kano-QFD research uses traditional industry products as the research object, and research on the interaction design of digital products is rare. In particular, research that focuses on ICH apps is lacking, and most such studies focus on interface design, with the UX design of handicraft ICH apps remaining underexplored.

Therefore, this research combines user needs research with handicraft ICH app design and introduces Kano-QFD to explore the UX design of handicraft ICH apps.

## Research design

**Fig. 2:**
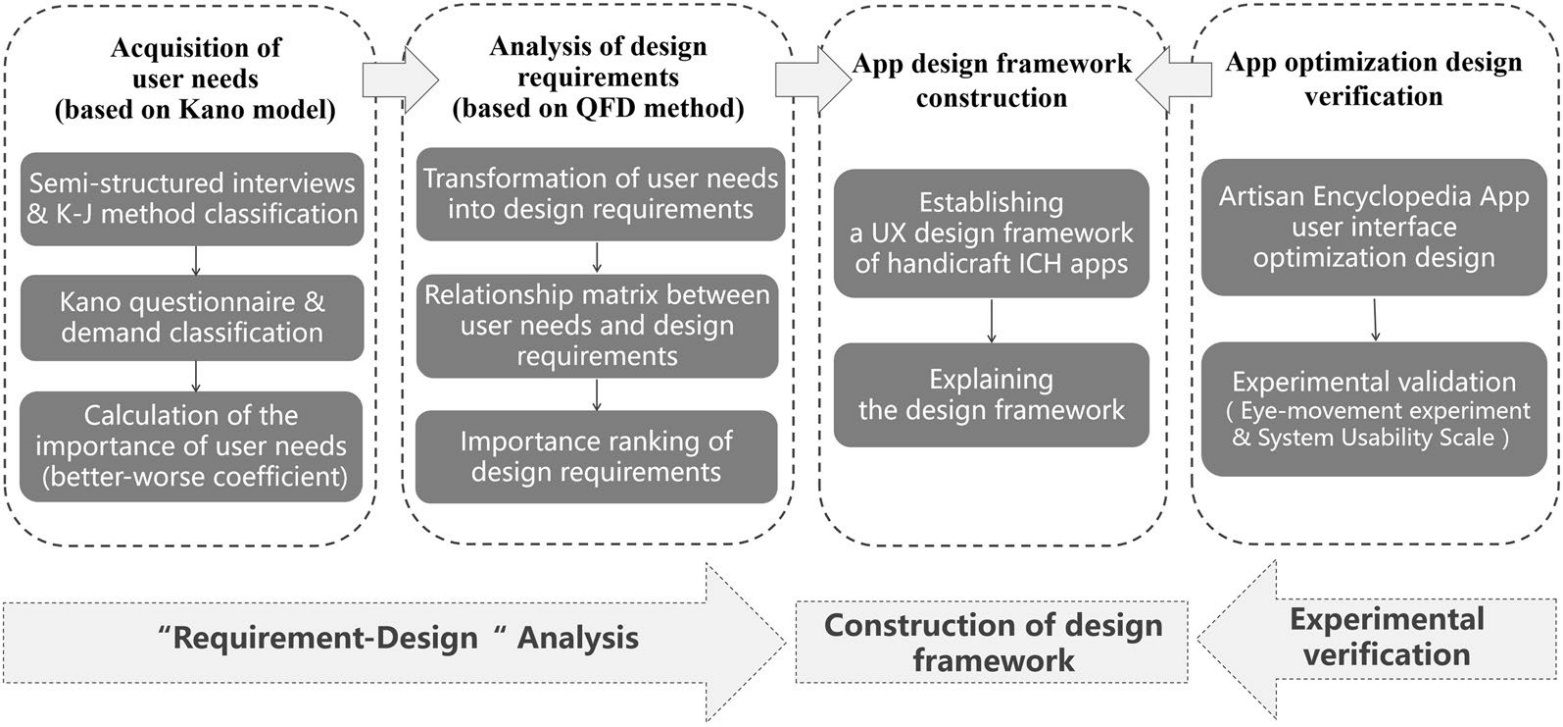
Research flowchart

A UX design study of handicraft apps was conducted. First, user needs data were collected via semi-structured interviews with experienced handicraft ICH app users. User needs were then classified using the K–J method which organizes ideas into relationships [44]. Next, a Kano questionnaire was combined with Kano classification to quantitatively analyze those user needs, and their importance was calculated. The QFD method was then used to transform user needs into app design requirements and to establish a relationship matrix between user needs and design requirements. The app design requirements were then ranked in order of importance. A UX design framework of the handicraft ICH apps was then established based on the importance of the design requirements. Finally, the effectiveness of the design framework was verified through the optimized design of one handicraft ICH app (see Fig. 2 for the research flowchart).

### Kano model: user needs acquisition

Based on the user-centered concept, product development is directed design based on user needs [45]. User needs, therefore, are important indicators of the needs of target app users and serve as a reference for the design of the Kano questionnaire.

#### Semi-structured interviews

Key informant interviews [46] were conducted with eight participants (four men and four women) recruited from across China via snowball sampling. Owing to the COVID-19 pandemic and the varied locations of participants, all interviews were conducted online and each lasted approximately 20 min. New concepts were added following each interview and the interview schedule was amended and improved. No new problems emerged in the eighth interview and, therefore, no more interviews were required. The interviewees ranged in age from 21 to 69 years and all were experienced users of handicraft ICH apps. Each of the interviewees had been users of these apps for between 1 and 5 years (see Table 1 for participant information). The purpose of the interviews was to obtain information about user needs and the questions focused on functional experience, emotional experience, and environmental experience (see Additional file 1: Appendix A). The K–J method was then applied to organize and summarize the interview data. First, we converted the original videos of the interviews into written records. Second, the data were classified and summarized based on schema and similarity. Finally, we identified 12 user needs with core significance, which we defined as user needs that were relatively independent and could lead to the transformation from user needs to design requirements (see Table 2).

**Table 1:** Interviewee information

S/N	Occupation	Age	Education	Usage time	Characteristics
F01	Associate professor	52	Master’s Degree	Five years	Uses many types of handicraft ICH apps, especially puzzle games and decryption apps; regular and deep user of the Jiangmu* app
F02	Civil servant	38	Master’s Degree	One year	Active participant in social media and online communities; enjoys life and social apps such as kiinii app and Yitiao app
F03	University faculty	46	PhD	Three years	Conducts academic research on the use of handicraft ICH apps; prefers learning and education handicraft ICH apps; enjoys sharing experiences in interactive communities
F04	Corporate manager	47	Bachelor’s Degree	Two years	Runs an art institution; enjoys crafts and uses craft-related apps frequently; especially enjoys watching craft videos and interactive reviews; occasionally shops online
F05	Marketing specialist	43	Bachelor’s Degree	Five years	Fascinated by the dynamic and digital presentation of handicraft ICH apps; a deep user of the Sunmao app and the Zheshan app
F06	Self-media operator	32	Bachelor’s Degree	One year	Uses several categories of handicraft ICH apps; enjoys cultural communication apps; deep user of the Lanxiong Live app and has met many artisans and craftsmen through the platform
F07	International student	21	Undergraduate	Two years	In-depth experience with handicraft ICH apps because of his hobby; strongly recommends game handicraft ICH apps, such as Jiangmu app, his friends; orders favorite handicrafts from Huaxia Jiangren app
F08	Retired high school teacher	69	Bachelor’s Degree	One year	Enjoys lifestyle handicraft apps; browses designs; watches animations; often reads comments but rarely posts; enjoys goods and services handicraft apps; occasionally buys items

*App included and described in Additional file 1: Appendix B

**Table 2:** User needs of handicraft ICH apps

S/N	User needs	S/N	User needs	S/N	User needs
U1	Craft feature	U5	Interaction	U9	Immersion experience
U2	Content level	U6	Interface design	U10	Multidimensional perception
U3	Information push	U7	Cultural atmosphere	U11	Social demands
U4	Practical life	U8	Privacy guarantee	U12	Product service

Given the small sample size, we sought to ensure that the data were comprehensive and representative by conducting triangulation with app user reviews and the findings of academic research. First, we compared the interview data with online user reviews of handicraft ICH apps. We used 82 handicraft ICH apps available for download from the iOS App Store and the Android Huawei App Market. The apps were located using a keyword search and associated app recommendations (until October 26, 2022). The apps were divided into five categories, according to platform attributes and content, including cultural communication, life and society, learning and education, entertainment and games, and commercial services. Using the App Store list ranking and the Huawei App Market downloads as standard, three apps were selected in each category, and differences in user satisfaction were identified from the reviews (see Additional file 1: Appendix B). Reviewers discussed problems with content, interaction, aesthetics, and security. These comments were random and scattered and, in the case of some apps, few or nonexistent. While it was difficult to comprehensively and systematically understand user needs based on reviewer comments, they provided some triangulation support for the findings of the semi-structured interviews.

Second, we conducted a keyword search of the China National Knowledge Internet (CNKI), a database of Chinese academic and government publications, using the keywords “handicraft intangible cultural heritage (ICH) apps & user needs” or “traditional handicraft apps & user experience (UX) design” for the time period 2018–2022. There were few relevant papers on this topic prior to 2018 and the reference value was limited. We obtained 13 papers (see Additional file 1: Appendix C) and compared the user needs listed in these publications with those of the interviewees.

These comparisons revealed that the 12 user needs arising from the interviews are representative and comprehensive.

#### Kano questionnaire and demand classification

The Kano questionnaire sought to establish the importance of the 12 user need requirements. The questionnaire consisted of two questions for each requirement—one positive (i.e., when the requirement is realized) and one negative (i.e., when the requirement is not realized). The questions were asked according to a 5-point Likert scale [47, 48], consisting of “satisfied”, “should be so”, “does not matter”, “acceptable”, and “dislike” (see Additional file 1: Appendix D).

The Kano questionnaire was administered via a random survey of internet users. Seventy-seven questionnaires were released via Wenjuanxing, a Chinese online survey software platform. Seventy-four valid questionnaires were returned, and three invalid questionnaires were omitted (these were completed in less than 50 s and contained identical answers to each question), with an effective rate of 96.1%. The percentage of males and females was 47.3% and 52.7%, respectively. The participants covered a range of ages, with 10.81% aged below 18 years, 60.81% aged 19–45, 14.86% aged 46–65, and 13.51% aged above 65. Participants were asked about experience using handicraft ICH apps, and 39.19% reported that they used such apps and 60.81% that they did not. SPSSAU statistical software was used to conduct reliability analysis of the valid questionnaires, and the Cronbach α coefficients of the forward questions and the reverse questions were 0.793 and 0.927, respectively, demonstrating good internal consistency and reliable survey results. KMO values were 0.736 and 0.855, respectively, and the cumulative variance contribution rates were 62.081% and 69.680%, respectively, showing that the questionnaire had good structural validity and the research item information could be effectively extracted.

Next, the Kano model classification of each user’s needs was determined based on the questionnaire results (Table 3).

**Table 3:** User requirement assessment using the Kano model [35]

User requirements	Not implemented				
	Satisfied	Should be so	Does not matter	Acceptable	Dislike
*Implemented*					
Satisfied	Q	A	A	A	O
Should be so	R	I	I	I	M
Does not matter	R	I	I	I	M
Acceptable	R	I	I	I	M
Dislike	R	R	R	R	Q

Attractive quality (A), One-dimensional quality (O), Must-be quality (M), Indifferent quality (I), Reverse quality (R), and questionable (Q)

The Kano classification of user needs was determined according to the maximum frequency of A, O, M, I, and R. If there were multiple identical Kano category percentages for the same user needs, the classification was determined according to the influence of the Kano categories, that is, M > O > A > I [49]. The Kano model classification of user needs U1–U12 for the handicraft ICH apps was obtained (Table 4).

**Table 4:** User needs Kano classification and user needs importance

User needs	Kano attribute category						Final category	SII%	DDI%	Satisfaction% (C)	Importance (C_j_)
	A%	O%	M%	I%	R%	Q%					
U1	5.41	4.05	45.95	41.89	0.00	2.70	M	9.72	− 51.39	51.39	56.53
U2	6.76	4.05	47.30	40.54	0.00	1.35	M	10.96	− 52.05	52.05	57.22
U3	12.16	2.70	39.19	43.24	2.70	0.00	I	15.28	− 43.06	43.06	43.06
U4	16.22	8.11	41.89	32.43	1.35	0.00	M	24.66	− 50.68	50.68	55.75
U5	25.68	41.89	12.16	18.92	1.35	0.00	O	68.49	− 54.79	68.49	82.19
U6	21.62	37.84	12.16	24.32	2.70	1.35	O	61.97	− 52.11	61.97	74.36
U7	14.86	9.46	39.19	31.08	4.05	1.35	M	25.71	− 51.43	51.43	56.57
U8	18.92	36.49	22.97	17.57	2.70	1.35	O	57.75	− 61.97	61.97	74.36
U9	47.30	17.57	8.11	25.68	0.00	1.35	A	65.75	− 26.03	65.75	92.05
U10	29.73	36.49	8.11	22.97	1.35	1.35	O	68.06	− 45.83	68.06	81.67
U11	16.22	8.11	40.54	33.78	0.00	1.35	M	24.66	− 49.32	49.32	54.25
U12	9.46	5.41	29.73	50.00	4.05	1.35	I	15.71	− 37.14	37.14	37.14

Attractive quality (A), One-dimensional quality (O), Must-be quality (M), Indifferent quality (I), Reverse quality (R), and questionable (Q)

#### User needs importance calculation

User satisfaction was calculated using a better-worse coefficient with the formulas SII = (A + O)/(A + O + M + I) and DDI = − (O + M)/(A + O + M + I). The user satisfaction index (C) is calculated by integrating the two: C = max(|SII||DDI|) [50]. The absolute value of SII and DDI is between 0 and 1. The importance level of user needs is lower when the value tends toward 0 and is higher when it tends toward 1.

To further accurately determine the importance of user needs (C_j_), an adjustment coefficient was introduced to the user satisfaction index (C). The adjustment coefficient for A was 0.4, for O 0.2, for M 0.1, and for other qualities 0 [51]. Note that I did not affect user satisfaction, so U3 and U12 were not included in the analysis. The importance of user needs (C_j_) is shown in Table 4.

### QFD method: analysis of design requirements

The QFD method transforms user needs into reasonable design requirements, establishes the relationship matrix between demand elements and design requirements, quantifies the relationship between user needs and design requirements using the House of Quality, and prioritizes design requirements [52, 53].

#### Translation of user needs into design requirements

A panel of five experts was invited to deliberate over the translation from user needs to design requirements (see Additional file 1: Appendix E). All five have industry experience, and four are academics. Two served as judges for the Internet Creativity and Entrepreneurship competition and the Service Design competition, while two experts founded a design agency, led design teams, and have completed a number of digital interaction design projects.

Using the mapping relationship method to map one-to-one or one-to-many correspondence between user requirements and design requirements, the panel of experts transformed the acquired handicraft ICH apps user needs into design requirements, and the mapping points of the transformation of user needs were explored in multiple directions. After receiving the reports from the panel, we summarized and organized the data, and obtained 10 primary design requirements and 23 secondary design requirements (Table 5).

**Table 5:** Transformation of user needs to design requirements

User needs		Design requirements			
		Primary design requirements		Secondary design requirements	
U1	Craft feature	D1	Introduction of handicraft ICH	D02	ICH stories and context
				D03	Inheritors and masterpieces
				D08	Links to ICH resources
U2	Content level	D2	Content section	D12	Difficulty mode
				D16	Creation and release of works
U4	Practical life	D3	Technique workshop	D19	Customized products
				D20	Precise information pushing
U5	Interaction	D4	Interactive operation	D24	Interactive guide tips
				D25	Scene interaction
				D26	Help center
U6	Interface design	D5	Interface visual design	D29	Information classification
				D30	Interface navigation
				D32	Dynamic/Static design
U7	Cultural atmosphere	D6	Cultural aesthetics	D38	Vocal design
				D39	Cultural icons
U8	Privacy guarantee	D7	Privacy and security	D41	Information protection
				D42	Account security
U9	Immersion experience	D8	Immersive experience	D46	Virtual game
U10	Multidimensional perception	D9	Smart Space	D49	Digital scene
				D50	Smart voice
U11	Social demands	D10	Community service	D52	Artisan online
				D55	Craft community/forums
				D56	Live sharing

#### Needs-design requirements relationship matrix

The relationship between user needs and design requirements is quantitatively reflected in the House of Quality, with the user needs elements on the left side of the house, the design requirements elements on the roof, and the relationship values between user needs and design requirements in the middle of the house. The House of Quality was scored by the expert panel. The symbols ◎, ○, and △ were used to indicate a strong correlation, general correlation, and weak correlation, respectively. According to the criteria ◎ = 5, ○ = 3, and △ = 1 [54]. The calculation values were expressed as R_ij_ (i.e., the value of the relationship between the i-th user’s needs and the j-th design requirements), as shown in Table 6.

**Table 6:** User needs and design requirements relationship matrix

User needs	C_j_	D1			D2		D3		D4			D5			D6		D7		D8	D9		D10		
		D02	D03	D08	D12	D16	D19	D20	D24	D25	D26	D29	D30	D32	D38	D39	D41	D42	D46	D49	D50	D52	D55	D56
U1	56.53	◎	◎	Ο		△	△									△					△	◎	Ο	△
U2	57.22				◎	Ο		△				△												
U4	43.06						◎	◎									△	Ο	△					△
U5	55.75								◎	◎	△								Ο	Ο	Ο			
U6	82.19				△					△		◎	Ο	◎	Ο	Ο			Ο	Ο	Ο			
U7	74.36	Ο	△											△	◎	◎			Ο			△		Ο
U8	56.57					Ο		△									◎	◎	△					
U9	74.36				△														◎	Ο	△			△
U10	92.05									Ο				Ο					Ο	◎	◎		△	
U11	81.67						△	△														Ο	◎	Ο

Strong correlation (symbol: ◎), General correlation (symbol: Ο), and Weak correlation (symbol: △)

#### Order of importance of design requirements

$$W_j = \sum_{i = 1}^m {\text{C}}_{j} {\text{R}}_{ij}\, \left(j = 1,2,3,\ldots,n\right)$$

Using the above formula, based on R_ij_ calculated in Table 5 and combined with the importance of the user requirements (C_j_), we calculated the importance of each design requirement (W_j_). The design items were then sorted in order of importance, as shown in Table 7.

**Table 7:** Importance ranking of design requirements

Design requirements	D02	D03	D08	D12	D16	D19	D20	D24	D25	D26	D29	D30	D32	D38	D39	D41	D42	D46	D49	D50	D52	D55	D56
Importance (W_j_)	506	357	170	443	398	354	411	279	637	56	468	247	761	618	675	326	412	1384	1097	1005	602	670	642
Sort	11	17	22	13	16	18	15	20	8	23	12	21	4	9	5	19	14	1	2	3	10	6	7

As shown in Table 6, virtual game (D46), digital scene (D49), smart voice (D50), dynamic/static design (D32), cultural icons (D39), craft community/forums (D55), live sharing (D56), scene interaction (D25), vocal design (D38), artisan online (D52), and ICH stories and context (D02) were highly weighted. These design requirements should be the key content of handicraft ICH app design.

## Design framework construction

**Fig. 3:**
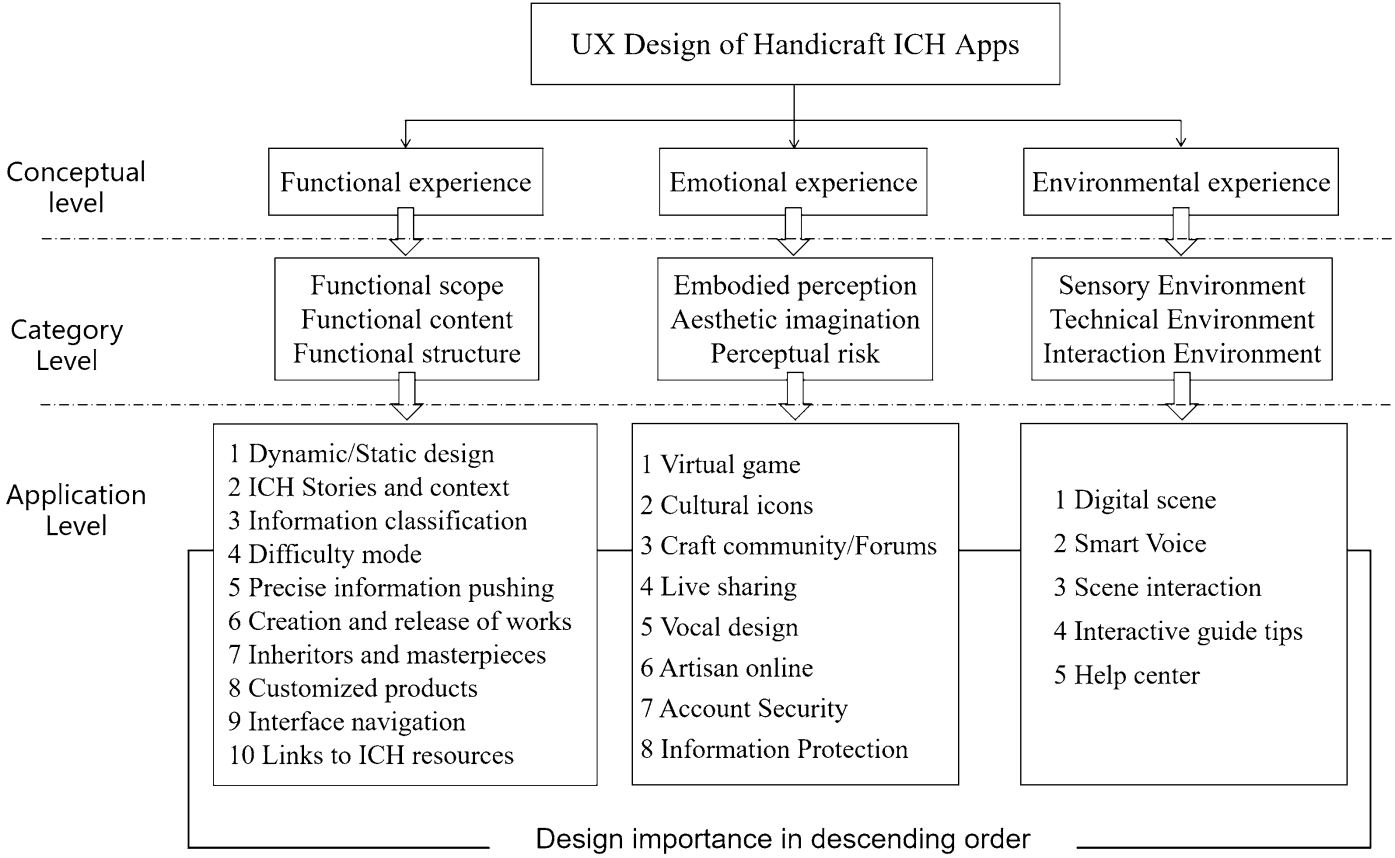
concept–category–application UX design framework for handicraft ICH apps

Based on our analysis and research, a UX design framework for handicraft ICH apps based on Kano-QFD, i.e., a concept–category–application UX design framework, was established (Fig. 3).

At the conceptual level, the user experience design of handicraft ICH apps is a combination of the three dimensions of functional experience, emotional experience, and environmental experience. These form the theoretical basis of app design. At the category level, users have diverse needs with regard to their interactive app experience, so user needs must be explored on multiple directions, which is the premise of app design. At the application level, the results show that each user need is transformed into specific design requirements and that the important design items are clarified in order of importance. Finally, different categories of handicraft ICH app are designed to optimize user experience.

## Experimental verification

We made a before-and-after comparison of UX optimization of the Artisan Encyclopedia app to verify the reliability of the concept–category–application UX design framework. In the experimental verification stage, we used the eye-movement experiment and System Usability Scale (SUS) to detect the UX effect of app interface optimization.

**Fig. 4:**
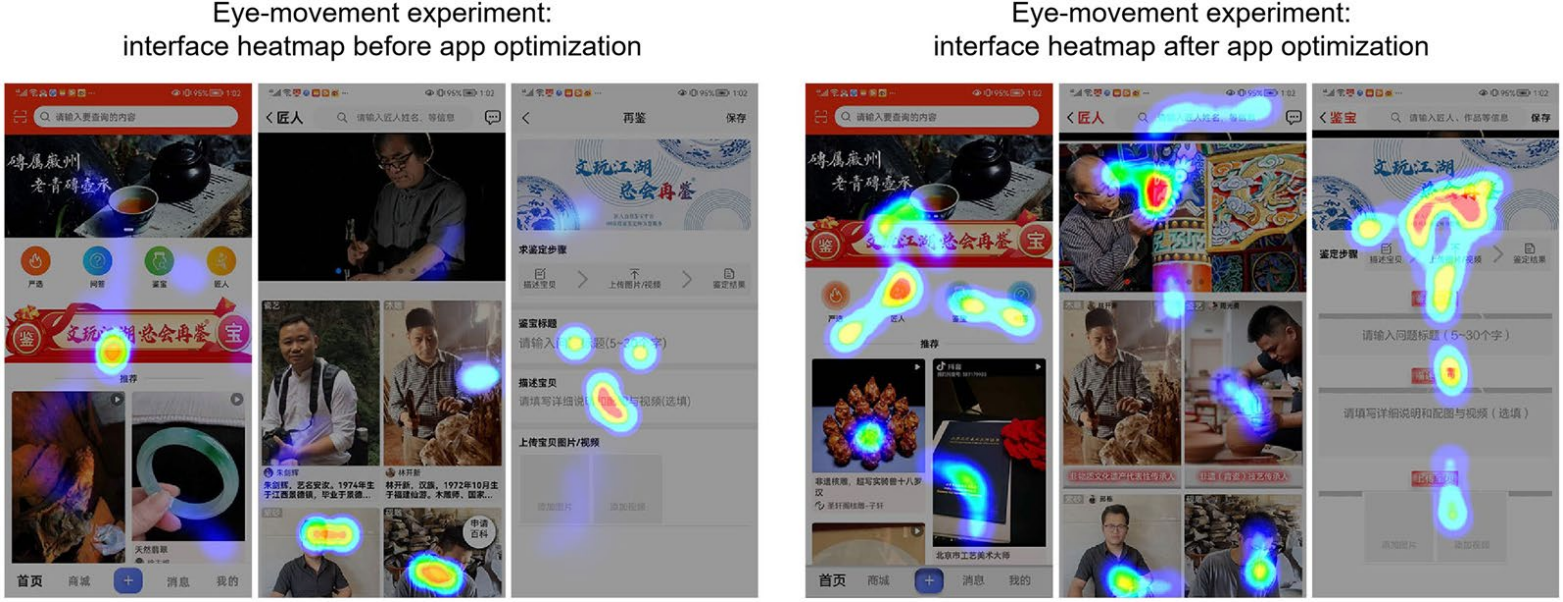
Artisan Encyclopedia app interface heatmap comparison

The eye-movement experiment is based on the eye gaze and eye-hopping activity of test subjects to record their visual activity. This adequately reflects visual flow and important attention points [42]. Three Artisan Encyclopedia app interfaces before and after optimization were selected as the control group and the experimental group, respectively, and the experiment was conducted using the Hawkeye Testing app from the iPhone App Store. Using this app, we were able to conduct eye tracking tests without the need for extra hardware. Eight users were randomly invited to participate as experimental subjects on a 1:1 ratio of use and no use experience, i.e., four participants had experience using the Artisan Encyclopedia app and four had no such experience. The experiment took place in the design studio of the Department of Industrial Design, Silla University, Korea. Using the Hawkeye Testing app, each participant observed the different interfaces for eight seconds each. The app automatically recorded eye movement data and generated an eye movement track and an eye-tracking heat map (Fig. 4). A comparison of the distribution of interest areas in the heatmaps before and after optimization revealed that the optimized visual process was smoother and that the focus was more prominent. The optimized interface had a better combination of dynamic and static design, and the icon design had a greater sense of digital scenes, demonstrating that the interface partition and information interaction of the optimized design interface were strongly improved, thus verifying the credibility of the "concept–category–application" UX design framework.

The System Usability Scale (SUS) is a satisfaction survey that assesses users’ perceptions based on their subjective emotions. The scale consists of 10 questions, with five questions each for positive and negative statements, as shown in Additional file 1: Appendix F. The eight subjects were scored on a 5-point scale for the two groups (based on three interfaces each), before and after optimization. The transformed scores for all question items were summed and multiplied by 2.5 (i.e., incremental factor) to obtain the total SUS score. The curve grading range of SUS scores is shown in Table 8.

**Table 8:** Curve grading range of SUS scores

Scores	84.1–100	80.8–84	78.9–80.7	77.2–78.8	74.1–77.1	72.6–74	71.1–72.5	65–71.0	62.7–64.9	51.7–62.6	0–51.7
Rating	A+	A	A−	B+	B	B−	C+	C	C−	D	F
Percentile	96–100	90–95	85–89	80–84	70–79	65–69	60–64	41–59	35–40	15–34	0–14

In comparison with Table 7, the interface conversion score of the Artisan Encyclopedia app before optimization was 68.2, with a rating of C; after optimization, the interface conversion score was 83.6, with a rating of A. The optimized interface score was greatly improved, further verifying the reliability of the "concept–category–application" UX design framework.

## Discussion and conclusions

Handicraft artisans and craftspeople are increasingly taking advantage of digital information technologies to disseminate their skills and expertise, market their products and attract a wider range of ICH inheritors. While cultural and societal changes continue to threaten ICH inheritance and protection, the use of social media and apps offers new opportunities. However, the success of these digital technologies depends on how well they meet user needs and the resulting user experience. Ongoing development and improvement of apps is critical for meeting the changing and more sophisticated needs of users and understanding how to use new media platforms to enhance public recognition and experience of handicraft ICH is essential to the protection and continued inheritance of ICH.

This study used semi-structured interviews and questionnaire surveys to introduce the Kano-QFD research method into UX design research in the area of handicraft ICH apps. It established the relationship matrix between user needs and design requirements, and then identified the importance of app design requirements. A UX design framework for handicraft ICH apps was established. The research clarified the three-level design framework of "concept–category–application" and verified the reliability of the design framework using an eye-movement experiment and the System Usability Scale.

The design framework ("concept–category–application") that we propose is important for the theoretical construction of handicraft ICH apps UX and, ultimately, for the design of internet products. In this study, Kano-QFD combined handicraft ICH app user needs with UX design to create new pathways for user needs research related to ICH digital products. From a practical perspective, the Kano-QFD method offers a new UX pathway for handicraft ICH practitioners and for app developers to better understand user needs and to develop apps that meet those needs in order of importance.

The purpose of this study was to develop theories concerning UX influencing factors in order to inform design practice. Actual design steps need to be determined according to the design of each handicraft ICH app. In addition to the impact of user experience, designers of handicraft ICH apps should also comprehensively consider the interests of ICH inheritors, brand owners, managers, developers, service providers, and other stakeholders and, with these in mind, build a blueprint for handicraft ICH app design based on user experience.

This research has a number of limitations. First, the initial investigation only included eight interviewees. However, the interview responses were triangulated against online app user reviews and the findings of limited existing research. Given time and sampling restriction, the study only targeted Chinese users and materials, and a long-term, follow-up survey is lacking. Therefore, future research should expand out from this base to include a larger sample of handicraft ICH users from different regions of China and beyond and include a broader range of app products. Such expanded research will contribute to the advancement of a broader range of UX designs for ICH digital products. Second, sampling for the Kano questionnaire was random rather than targeted. This approach was taken in the hope of obtaining more extensive user feedback. However, we overlooked the disjuncture between the simplicity of the Kano questionnaire and the diversity of users. Although users who had not previously used a handicraft ICH app could complete the questionnaire, the validity of their answers was not guaranteed. In order to acquire more meaningful data, future research should target experienced handicraft ICH app users. Finally, a long-term, follow-up investigation should be conducted on the uptake and use of representative handicraft ICH apps to verify the theory and obtain practically meaningful results.

## Supplementary Information

**Additional file 1.** Initial Interview Outline.

## Data Availability

Not applicable.
