# Supplementary material for: Kano-QFD-based analysis of the influence of user experience on the design of handicraft intangible cultural heritage apps
Source: Herit Sci. 2023 Mar 23;11(1):59. doi: 10.1186/s40494-023-00903-w (PMC10033176; doi:10.1186/s40494-023-00903-w)
Supplement: Supplementary file 1 — Additional file 1. Initial Interview Outline. [file 40494_2023_903_MOESM1_ESM.docx]

**Appendix A Initial Interview Outline**

1. What handicraft-related apps have you used and what are your reasons for using them?

2. What are your good and bad experiences of downloading, logging in, and using handicraft ICH apps?

3. What kind of content attracts you? What problems do you think should be avoided in the content?

4. Which formats do you think are most suitable for handicraft ICH apps (e.g., text, pictures, music, video, H5, or games)?

5. What characteristics should the interface design of handicraft-related apps have?

6. Is the aesthetic experience of using the app important? If yes, what aspects affect your aesthetic experience?

7. Are privacy protection and security tips important? If yes, what aspects of the experience design can be shown in the handicraft ICH apps?

8. Should the personalized services of handicraft ICH apps be adjusted according to different environments? If so, which services do you think are memorable?

9. What digital and intelligent technologies affect the use of handicraft ICH apps? Which technologies do you expect to see having greater involvement in handicraft ICH apps?

10. How much influence does product interaction have on app usage? Which aspects need to be focused on?

**Appendix B Handicraft ICH apps and user reviews**

(App Store ranking or Huawei App Market downloads as the standard; each category lists three applications)

| **Category** | **Platform** | **Introduction** | **User Comments** |
| --- | --- | --- | --- |
| Cultural communication | Artisan Encyclopedia  (匠人百科) | China's largest artisan database, an encyclopedic platform for artisans (artists, craftsmen); 22,000+ downloads. | Few user reviews, but overall good reviews. |
|  | Paper-cut Art Exhibition VR  剪纸艺术展VR | A digital online museum containing representative paper-cut works from seven provinces in China; 10,000+ downloads. | No user comments. |
|  | Lanxiong Live  (兰雄直播) | Folk culture and craft culture live broadcast platform, integrating short videos and live broadcasts; 23,000+ downloads. | Few user reviews, but overall good reviews. |
| Life and social | kiinii | A slow life interest community based on crafts and design, allowing traditional crafts to embrace modern design; 760,000+ downloads. | Negative reviews include cannot bind WeChat login and platform runs unstable. Positive reviews include ease of flashback when publishing personal works in the community. |
|  | Yitiao  (一条) | Highly influential lifestyle video and online trading platform for artisan crafts; 10 million+ downloads. | Negative reviews include the new online selection transaction experience feels bad. Positive reviews include the user evaluation is generally good. |
|  | Huaxia Fengwu  (华夏风物) | An online community interaction platform covering ICH skills and food specialties; 120,000+ downloads. | Negative reviews include errors in user content community uploads. User evaluation is generally good. |
| Learning and education | Sunmao  (榫卯) | Interactive form of learning 3D modeling decomposition of mortise and tenon structure; 350,000+ downloads. | Negative comments include the sense of interaction is not strong, the finger-sliding design of the page is not good, and the black dot sliding in the interface’s background makes people uncomfortable. |
|  | Feiyi Tongzefang  (非遗童泽坊) | A cultural and educational brand linked online and offline with ICH inheritors and professional scholars serving as mentors; 1,000+ downloads. | No user comments. |
|  | Jinse  (锦色) | An online coloring application in the folk New Year picture book style that provides a variety of graffiti painting tools; 75,000+ downloads. | Negative comments include the painting tools and colors are too limited, so playability is not high; customer service cannot be contacted; user's account is not available even though the user has successfully topped up. |
| Entertainment and games | Zheshan  (折扇) | 3-D presentation of the folding fan form, allowing users to experience the culture of folding fan craft as games; 750,000+ downloads. | Negative comments include short game process, lack of interaction and fun. User evaluations are generally good. |
|  | Jiangmu  (匠木) | Tenon and mortise-themed Chinese puzzle game, with space decryption tasks as the core gameplay; 3 million+ downloads. | Negative reviews include the game level free less. Overall user experience is good. |
|  | Piying: Nezha  (皮影: 哪吒) | A shadow culture game with shadow characters cutting paper, singing songs, and interacting with users; 2,000+ downloads. | Negative comments include the story version is imperfect; the interface artwork is rough; there is gameplay only. |
| Commercial services | Huaxia Jiangren  (华夏匠人) | A mobile e-commerce platform for crafts customization and trading for master craftsmen and artisans; 130,000+ downloads. | User evaluation is generally good. |
|  | Feiyi Guanjia  (非遗管家) | A platform for ICH transactions, ICH inheritor recommendations, and the promotion of traditional culture; 12,000+ downloads. | No user comments. |
|  | Mei Feiyi  (美非遗) | An online ICH mall covering traditional skills and traditional arts; 1,000+ downloads. | Low rating, no user reviews. |

Note: Data as of 19:00 on 26 October 2022

**Appendix C Academic papers searched on CNKI (2018–2022)**

(P01–P13 in chronological order of publication)

| **S/N** | **Literature** | **Author** | **Publication** | **Date** |
| --- | --- | --- | --- | --- |
| P01 | Research on the Dissemination and Promotion of Traditional Handicraft Intangible Cultural Heritage Mobile Apps in the Context of Digitization  数字化背景下传统技艺类非遗手机 App传播与推广研究 | Gong CY   1. 龚春英 | 1. Journal of Changzhou Institute of Technology 2. 常州工学院学报 | 2022 |
| P02 | Intangible Cultural Heritage APP Interface Design Based on Conjoint Analysis  基于联合分析法的非遗文化APP界面设计 | Ren YL, Chang H, Gu YS  任英丽, 常虹, 谷岩帅 | Packaging Engineering  包装工程 |  |
| P03 | Preliminary Study on the Design of Wide Color Porcelain App from the Perspective of Service Design  服务设计视阈下广彩瓷App设计初探 | Li XY, Zhang XY  黎欣悦, 张轩瑜 | Idea & Design  创意设计源 |  |
| P04 | Study on the Productive Protection Mode of Longchang Chabu Intangible Cultural Heritage from the Perspective of Service Design  服务设计视域下隆昌夏布非遗生产性保护模式研究 | Liu LL, Chen FH, Song H  刘玲玲, 陈凤虎, 宋好 | Hunan Packaging  湖南包装 |  |
| 1. P05 | 1. APP Architecture Design with the Theme of Inheritance and Innovation of Guangdong Intangible Cultural Heritage Skills以广东非遗技艺传承创新为主题的APP架构设计 | 1. Chen LN 2. 陈丽娜 | 1. China New Telecommunications 2. 中国新通信 | 2021 |
| 1. P06 | 1. E-commerce Promotion Strategy of Local Traditional Handicraft - Take "Mianzhou Handicraft" App as an Example 2. 地方传统手工技艺的电商推广策略--以“绵州手艺”App为例 | 1. Hu YS, Li YL, Li YF 2. 胡雨诗, 李友琳, 李怡芳 | 1. Market Modernization 2. 商场现代化 |  |
| 1. P07 | 1. Research on Interactive Design of Dong Embroidery APP Based on User Experience 2. 基于用户体验的侗族刺绣APP交互设计研究 | 1. Huang TY 2. 黄天瑶 | 1. Journal of Hubei Industrial Polytechnic 2. 湖北工业职业技术学院学报 |  |
| 1. P08 | 1. Beichuan "Qiangshan Queshe Tea" APP Interface Design in the Perspective of Intangible Cultural Heritage 2. “非遗”视域下的北川“羌山雀舌茶”APP界面设计研究 | 1. Pan HL, Li J 2. 潘红莲, 李杰 | 1. Art Research 2. 艺术研究 | 2020 |
| 1. P09 | 1. New challenges and Countermeasures of digital communication of China's intangible cultural heritage 2. 基于用户体验的非遗 APP 设计与应用研究 | 1. Zhang T, Peng L 2. 张婷, 彭莉 | 1. Packaging Engineering 2. 包装工程 |  |
| 1. P10 | 1. Research on Intangible Cultural Heritage Handicraft APP Design Based on Service Design 2. 基于服务设计的非遗手工艺APP设计研究 | 1. Xu L, Lu Y 2. 许莉, 路由 | 1. Design 2. 设计 |  |
| 1. P11 | 1. Research on APP Design of Intangible Cultural Heritage Community Based on User Experience 2. 基于用户体验的非遗社区APP设计研究 | 1. Yang LQ, Gao Z 2. 杨利强, 高瞩 | 1. Design 2. 设计 |  |
| 1. P12 | 1. Interaction Design of Traditional Handicraft Type APP Based on Cognitive Schema 2. 基于认知图式的传统手工艺类APP交互设计 | 1. Chen GY, Ji Y, Tan P 2. 陈国盈, 纪毅, 檀鹏 | 1. Packaging Engineering 2. 包装工程 | 2019 |
| 1. P13 | 1. APP Product Architecture Design Focus on the Guangxi Zhuang Bronze Drum Culture 2. 以广西壮族铜鼓文化传播为主题的APP产品架构设计 | 1. Zhang T, Chen GX 2. 张婷, 陈光喜 | 1. Packaging Engineering 2. 包装工程 | 2018 |

**Appendix D Questionnaire survey on the needs of handicraft ICH app users**

Thank you very much for taking the time to fill out the questionnaire! We are researching the design of a handicraft intangible cultural heritage (ICH) app and would like to know about your user needs. Please tick the box for confirmation. The data obtained will be used for research only. Thank you for your cooperation and wish you a happy life!

Options Description: The following are five specific criteria for satisfaction selection. Please choose after comparison.

- Satisfied: Feeling satisfied and surprised.
- Should be so: Deserves to exist, is a must.
- Doesn't matter: It does not matter whether or not you like it.
- Acceptable: Not necessarily like, but still acceptable.
- Dislike: Feeling dissatisfied and unable to accept.

Gender: Male Female

Age: 0–18 19–30 31–45 46–65 65+

Have you used any handicraft ICH apps: Yes No

| **Positive Questions** | | | | | | |
| --- | --- | --- | --- | --- | --- | --- |
| **S/N** | **Content** | **Handicraft ICH apps user needs satisfaction** | | | | |
|  |  | **Satisfied** | **Should be so** | **Doesn't matter** | **Acceptable** | **Dislike** |
| 1 | The app is distinctive in the theme of "handicraft". |  |  |  |  |  |
| 2 | The app distinguishes different levels of difficulty in terms of content. |  |  |  |  |  |
| 3 | The app has personalized content pushing. |  |  |  |  |  |
| 4 | The app content is practical in life. |  |  |  |  |  |
| 5 | The app is easy to operate and highly interactive. |  |  |  |  |  |
| 6 | The app interface is aesthetically pleasing and consistent in style. |  |  |  |  |  |
| 7 | The app has a cultural atmosphere with traditional crafts. |  |  |  |  |  |
| 8 | The app has information security and privacy protection. |  |  |  |  |  |
| 9 | The app brings a sense of immersive experience. |  |  |  |  |  |
| 10 | The app has multi-dimensional perception, such as visual, auditory, and tactile senses. |  |  |  |  |  |
| 11 | The app provides social interaction such as activities, creation, and sharing. |  |  |  |  |  |
| 12 | The app provides product consumption and services such as shopping and education. |  |  |  |  |  |

| **Reverse Questions** | | | | | | |
| --- | --- | --- | --- | --- | --- | --- |
| **S/N** | **Contents** | **Handicraft ICH apps user needs dissatisfaction** | | | | |
|  |  | **Satisfied** | **Should be so** | **Doesn't matter** | **Acceptable** | **Dislike** |
| 1 | The app is not distinctive in the theme of "handicraft". |  |  |  |  |  |
| 2 | The app does not distinguish different levels of difficulty in terms of content. |  |  |  |  |  |
| 3 | The app does not have personalized content pushing. |  |  |  |  |  |
| 4 | The app content is not practical in life. |  |  |  |  |  |
| 5 | The app is difficult to operate and highly interactive. |  |  |  |  |  |
| 6 | The app interface is not aesthetically pleasing and inconsistent in style. |  |  |  |  |  |
| 7 | The app does not have a cultural atmosphere with traditional crafts. |  |  |  |  |  |
| 8 | The app does not have information security and privacy protection. |  |  |  |  |  |
| 9 | The app does not provide a sense of immersive experience. |  |  |  |  |  |
| 10 | The app does not have a multi-dimensional perception, such as visual, auditory, or tactile senses. |  |  |  |  |  |
| 11 | The app does not provide social interaction, such as activities, creation, and sharing. |  |  |  |  |  |
| 12 | The app does not provide product consumption and services such as shopping and education. |  |  |  |  |  |

**Appendix E Expert Panel Members**

| **S/N** | **Expert** | **Occupation** | **Age** | **Education** | **Characteristics** |
| --- | --- | --- | --- | --- | --- |
| F01 | Dr. Kang | University associate  professor | 46 | PhD | Main research area is internet product interaction design; studied product design at Silla University in Korea and was a visiting scholar at Michigan State University; has published more than 20 academic papers on user experience and design management; has been involved in many internet product development projects; is a judge of service design competitions and internet competitions. |
| F02 | Dr. Huang | University associate  professor | 42 | PhD | Head of the Department of Advertising Design at SISU; vice president of the Creative Industries Association; serves as a judge for internet creativity and entrepreneurship competitions; excels in user research and digital product design. |
| F03 | Dr. Tu | University  professor | 41 | PhD | Main research area is the modern communication design of traditional craft culture; has published more than 30 academic papers; leads an academic team; highly experienced in user and audience surveys. |
| F04 | Mr. Liu | Design Director | 37 | Master’s | Has taught in universities; published more than 10 relevant academic papers in academic journals; founded a design agency; led a design business team working on industrial product design, app design, branding, etc. |
| F05 | Mr. Zhao | Product Manager | 43 | Bachelor’s | Has worked for over twenty years in print media, corporate marketing planning departments and internet companies; specializes in product interaction design and creative communication; has led and participated in several internet product development projects; led a team to run self-publishing accounts. |

**Appendix F System Usability Scale (SUS)**

| **S/N** | **Content** | **Strongly disagree** | **Disagree** | **Neutral** | **Agree** | **Strongly agree** |
| --- | --- | --- | --- | --- | --- | --- |
| 1 | I would like to use this app frequently. |  |  |  |  |  |
| 2 | I found the app unnecessarily complex. |  |  |  |  |  |
| 3 | I thought the app was easy to use. |  |  |  |  |  |
| 4 | I need the support of an experienced person or technician to use the app. |  |  |  |  |  |
| 5 | I found the various functions of the app were well integrated. |  |  |  |  |  |
| 6 | I thought there was too much inconsistency in the app. |  |  |  |  |  |
| 7 | I thought most people would learn to use the app quickly. |  |  |  |  |  |
| 8 | I found the app cumbersome to use. |  |  |  |  |  |
| 9 | I felt confident using the app. |  |  |  |  |  |
| 10 | I needed to learn a lot before I could use this app. |  |  |  |  |  |
